# Parkin Mediates Apparent E2-Independent Monoubiquitination *In Vitro* and Contains an Intrinsic Activity That Catalyzes Polyubiquitination

**DOI:** 10.1371/journal.pone.0019720

**Published:** 2011-05-23

**Authors:** Katherine C. M. Chew, Noriyuki Matsuda, Keiko Saisho, Grace G. Y. Lim, Chou Chai, Hui-Mei Tan, Keiji Tanaka, Kah-Leong Lim

**Affiliations:** 1 Department of Physiology, National University of Singapore, Singapore, Singapore; 2 Laboratory of Frontier Science, Tokyo Metropolitan Institute of Medical Science, Tokyo, Japan; 3 A*STAR Duke-NUS Neuroscience Research Partnership, Singapore, Singapore; 4 National Neuroscience Institute, Singapore, Singapore; University of Groningen, The Netherlands

## Abstract

**Background:**

Mutations in the parkin gene, which encodes a ubiquitin ligase (E3), are a major cause of autosomal recessive parkinsonism. Although parkin-mediated ubiquitination was initially linked to protein degradation, accumulating evidence suggests that the enzyme is capable of catalyzing multiple forms of ubiquitin modifications including monoubiquitination, K48- and K63-linked polyubiquitination. In this study, we sought to understand how a single enzyme could exhibit such multifunctional catalytic properties.

**Methods and Findings:**

By means of *in vitro* ubiquitination assays coupled with mass spectrometry analysis, we were surprised to find that parkin is apparently capable of mediating E2-independent protein ubiquitination *in vitro*, an unprecedented activity exhibited by an E3 member. Interestingly, whereas full length parkin catalyzes solely monoubiquitination regardless of the presence or absence of E2, a truncated parkin mutant containing only the catalytic moiety supports both E2-independent and E2-dependent assembly of ubiquitin chains.

**Conclusions:**

Our results here suggest a complex regulation of parkin's activity and may help to explain how a single enzyme like parkin could mediate diverse forms of ubiquitination.

## Introduction

Mutations in the parkin gene are a predominant cause of autosomal recessive early-onset parkinsonism [1]. Further, emerging evidence also suggests a link between parkin expression variability and sporadic Parkinson's disease (PD) [2]. Accordingly, a better understanding of parkin function could help elucidate pathways underlying PD pathogenesis. Initial studies performed by three independent groups revealed that parkin functions as a ubiquitin ligase (E3) associated with the ubiquitin-proteasome system (UPS) [3], [4], [5], a major intracellular proteolytic machinery that destroys unwanted proteins. In this system, parkin as an E3 member collaborates with two other members, i.e. ubiquitin-activating (E1) and -conjugating (E2) enzymes, to catalyze the formation of a ubiquitin chain on its substrates that acts as a targeting signal for proteasome-mediated degradation. Typically, the ligation reaction associated with ubiquitin-mediated protein degradation occurs between the terminal residue (G76) of one ubiquitin molecule and an internal lysine (K) residue at position 48 within another. However, ubiquitin chain assembly can also occur at alternative K residues within the molecule, such as K63. In addition, proteins can also be monoubiquitinated [6], [7]. These non-canonical ubiquitin modifications usually serve as non-proteolytic signals involved in various cellular processes including DNA repair and endocytosis [7].

Although originally associated with protein degradation, we and others have demonstrated that parkin is a unique E3 capable of mediating monoubiquitination as well as K63-linked polyubiquitination [8], [9], [10], [11]. However, it is intriguing to note that a single enzyme could exhibit such multifunctional properties. An attractive speculation is that the choice of E2 partners could influence the topology of ubiquitin chain assembly mediated by parkin. Indeed, parkin interaction with the heterodimeric Ubc13/Uev1a E2 pair appears to favor K63-linked polyubiquitination whereas its partnership with UbcH7 promotes K48-linked polyubiquitination [8], [12]. Notwithstanding this, the determinants that regulate parkin's choice of E2 remain unknown although a recent study suggested that parkin phosphorylation by PINK1 facilitates its recruitment of Ubc13 [13]. Furthermore, the differential recruitment of E2s by parkin cannot adequately explain its monoubiquitination activity.

Here, we demonstrated that parkin is a unique E3 capable of mediating ubiquitination in an E2-independent manner, a novel activity by an E3 member that is unprecedented. Interestingly, whereas full length parkin catalyzes E2-independent monoubiquitination *in vitro*, a truncated mutant retaining only the C-terminal IBR-R2 region catalyzes both mono and polyubiquitination in the absence of E2. Supporting this, mass spectrometry (MS) analysis revealed the presence of K48-linked polyubiquitin in reaction products catalyzed by IBR-R2 but not full length parkin. Importantly, ubiquitin chains formed by IBR-R2 become modified in the presence of E2s. For example, mixed chains of K48- and K63-linked ubiquitin polymers are generated when UbcH7 is replaced by Ubc13/Uev1a. No such modifications in the presence of E2s occur in reactions containing full length parkin. Taken together, our results suggest that parkin's IBR-R2 contains an intrinsic activity that catalyzes the formation of polyubiquitin chains in the absence or presence of E2s, and that this activity is masked in the full length protein, which catalyzes solely monoubiquitination *in vitro*.

## Methods

### Antibodies and reagents

MBP-parkin species containing Δ78, Δ152, or Δ237 truncations were generated by means of PCR using MBP-parkin as a template and subcloned into BamHI and EcoRI sites of pMAL-p2. Phospho-mimetic parkin S101D, S127D, S131D, T175D, T217D and S378D mutants were generated by site-directed mutagenesis using MBP-parkin (full length) as a template. The mutagenesis reactions were based on the QuickChange™ method (Stratagene). Other MBP-parkin constructs used were described previously [11]. TRAF6 cDNA was amplified from cDNA preparations isolated from HEK293 cells using the following primer pair: 5′-CGCGAATTCAGATGAGTCTGCTAAAC-3′ and 5′ - CGCCTCGAGCTATACCCCTGCATC - 3′, and subcloned into EcoRI and XhoI sites of pMAL-p2. Momo and Rma1 as well as RING finger domains of Trim5, Trim32, Rbx2, March8, Rnf4 and Deltex2 were subcloned by PCR and all recombinant MBP-fusion proteins were purified by the standard methods [11]. The following commercial antibodies were used: anti-ubiquitin FK1 and FK2 (BIOMOL), anti-E1 (BIOMOL), anti-parkin PRK8 (Covance) and anti-MBP (New England BioLabs). Anti-synphilin-1 was a kind gift from Dr. Engelender S (Technion-Israel). Unless, otherwise stated, all other reagents were purchased from Sigma.

### *In vitro* ubiquitination

The *in vitro* ubiquitination assay was performed essentially as described previously with slight modifications [11]. Briefly, purified MBP-tagged protein (20 µg/ml) was incubated in reaction buffer (50 mM Tris-HCl pH 8.8, 2 mM dithiothreitol, 5 mM MgCl_2_ and 2 mM ATP) together with 10 µg/ml recombinant human E1 (BIOMOL, unless otherwise stated), 10 µg/ml recombinant UbcH7 (BIOMOL) or purified Ubc13/Uev1a (4 ug/ml each) and 50 µg/ml ubiquitin (Calbiochem) for 2 h at 32°C. For synphilin-1 ubiquitination assay, 5 µg recombinant synphilin-1 was added into the reaction mix as a substrate for parkin. Equivalent volumes of post-reaction mix were boiled briefly before resolution by means of SDS-PAGE. The reaction products were analyzed by means of Western Blotting procedures using ECL detection reagents (Amersham). For MS analysis, the reactions were scaled up. In this case, 200 µg/ml purified MBP-tagged protein was incubated in reaction buffer (50 mM Tris-HCl pH 8.8, 2 mM DTT, 5 mM MgCl_2_ and 4 mM ATP) together with 20 µg/ml recombinant human E1 (BIOMOL), 20 µg/ml recombinant UbcH7 (BIOMOL) or purified Ubc13/Uev1a (8 ug/ml each) and 200 µg/ml ubiquitin (Calbiochem).

### MS analysis

The method for detecting specific linkages of polyubiquitin-derived tryptic digest via MALDI-TOF MS has been described previously [14]. Briefly, CBB-stained protein bands were excised from SDS-polyacrylamide, destained and in gel-digested with 10 µg/ml modified trypsin (Promega) in 20 mM ammonium bicarbonate. The resulting peptides were recovered, desalted and eluted with 0.5% TFA-50% acetonitrile before being spotted on analytical plates for MS analysis (4800 MALDI TOF/TOF, Applied Biosystems). MS and MS/MS data were analyzed by ProteinPilot software 2.0 (Applied Biosystems).

## Results

### Parkin mediates apparent E2-independent ubiquitination *in vitro*

**Figure 1:**
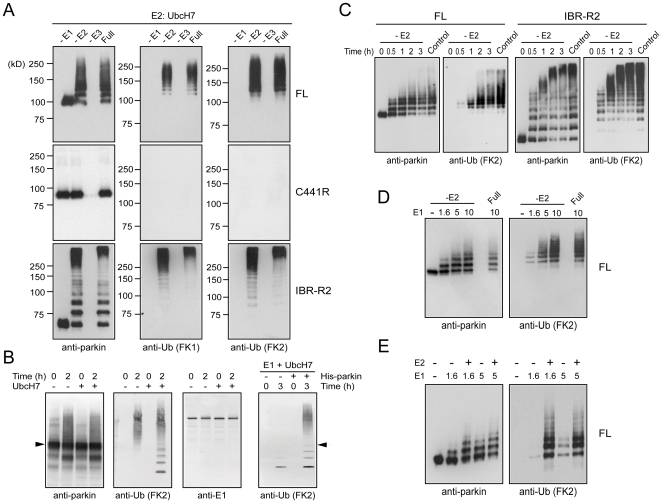
Parkin mediates self ubiquitination in the presence or absence of E2. (A) *In vitro* ubiquitination reaction products generated by MBP-parkin, C441R or IBR-R2 in the absence of E1, E2 or parkin (i.e. “-E3”), or in the presence of all three components (Full) were subjected to immunoblotting with anti-parkin, anti-FK1 and anti-FK2, as indicated. Notice the ladders of immunoreactivities observed in MBP-parkin and IBR-R2-catalyzed reactions but not in C441R-containing reactions. (B) Reaction products generated by His-tagged parkin (His-parkin) purified from insect cells in the presence or absence of UbcH7 at various time points were subjected to immunoblotting with anti-parkin, anti-FK2 and anti-E1, as indicated. Arrows indicate His-parkin. (C) Reaction products generated by MBP-parkin or IBR-R2 in the absence of E2 at various time points were subjected to immunoblotting with anti-parkin, anti-FK1 and anti-FK2. Control refers to full reactions. (D & E) MBP-parkin autoubiquitination assay was performed in the presence of different doses of E1 (i.e. 1.6, 5 or 10 µg/ml), in the presence or absence of E2 (as indicated) and visualized by means of anti-parkin and anti-FK2 immunoblotting.

MBP-parkin, -IBR-R2 and -C441R were purified according to recently published methods (Fig. S1A) and their activities in the presence of UbcH7 were independently assessed by means of a standard *in vitro* ubiquitination assay. Consistent with our previous results [11], we observed the presence of high molecular weight (HMW) parkin species derived from MBP-parkin self-ubiquitination in full reaction mixtures containing either full length parkin or IBR-R2 but not parkin C441R RING2 mutant (Fig. 1A). These HMW parkin species are not detected when E1 or parkin (referred to as “-E3” in figures) is omitted in the assay (Fig. 1A). Unexpectedly, in the absence of E2, both MBP-parkin and IBR-R2 appear capable of mediating autoubiquitination (Fig. 1A). Interestingly, FK1 immunoblotting, which specifically recognizes polyubiquitin species, generates a laddering pattern in MBP-parkin as well as IBR-R2 catalyzed reactions in the presence or absence of E2, although ubiquitinated species generated by IBR-R2 tend to be of higher molecular weight than those produced by the full length protein (Fig. 1A). FK2 immunoblotting, which recognizes both mono- and poly-ubiquitinated proteins, reveals a laddering pattern that is similar to FK1 (Fig. 1A). When compared side by side, both FK1 and FK2 immunoreactivities are significantly more robust in reactions catalyzed by IBR-R2 than those catalyzed by the full length protein (Fig. 4 & 5). In contrast, no FK1 or FK2 immunoreactivity is detectable in reactions conducted with parkin C441R mutant (Fig. 1A). Similar results were obtained with E1 enzyme from other commercial companies (Fig. S1B). Our results therefore suggest that parkin-mediated ubiquitination *in vitro* could occur in the presence or absence of E2 and that the nature of ubiquitination catalyzed by full length parkin and IBR-R2 probably differs.

To exclude the trivial possibility that E1 used in our reactions, although from different commercial preparations, may all be contaminated with trace amounts of E2s, we repeated our experiments with recombinant E1 purified from insect cells as well as from *E. coli*. Because *E. coli* is devoid of endogenous protein ubiquitination system, recombinant E1 purified from *E. coli* is absolutely free from contaminating E2s. We obtained essentially the same results with these highly pure E1 preparations (Fig. S1C). Similarly, baculovirus-expressed His-tagged full length parkin purified from insect cells also exhibits E2-independent auto-ubiquitination (Fig. 1B), suggesting that the phenomenon is not an artifact that has arisen from the fusion of an artificial substrate, i.e. MBP, to parkin. Importantly, in the absence of His-tagged parkin, E1 and E2 alone do not generate appreciable ubiquitin-positive bands (Fig. 1B). To extend on these findings, we also performed a time-dependent parkin ubiquitination assay in the absence of E2. Anti-parkin immunoblotting of the reaction products reveals an increase in the levels of oligomeric and HMW parkin species over time that correlate with a decrease in the level of monomeric parkin, suggesting progressive self ubiquitination (Fig. 1C). This is true for both MBP-parkin and IBR-R2-catalyzed reactions, except that the latter, is again apparently more active in mediating the formation of higher molecular weight parkin species (Fig. 1C). Notably, ubiquitinated reaction products generated by both MBP-parkin and IBR-R2 started to appear as early as half an hour after the start of reaction, suggesting that the observed E2-independent activity is unlikely a result of non-catalytic event (Fig. 1C). Taken together, our results demonstrate that parkin could mediate ubiquitination in the apparent absence of E2 *in vitro*.

Curiously, our previous characterization of MBP-parkin did not reveal its E2-independent activity [11]. We noted that a difference between our previous and current study is the concentration of E1 used, i.e. 1.6 and 10 µg respectively. To test if E1 concentration influences the E2-independent activity of recombinant parkin, we performed parkin ubiquitination assay in the presence of 1.6, 5.0 and 10 µg of E1. As expected, parkin-mediated E2-independent auto-ubiqiuitination increases in an E1 dose-dependent manner (Fig. 1D). Moreover, when E1 is kept at a low concentration (i.e. 1.6 µg), the presence of E2 markedly enhances parkin activity (Fig. 1E). This dependency on E2 by parkin is however diminished at higher concentrations of E1 (Fig. 1D & E). Our results thus suggest a relationship between E1 activity and parkin's E2 dependency and provide an explanation to the apparent discrepancy between our previous and current findings.

### E2-independent activity is specific to parkin and is dependent on RING2 integrity

**Figure 2:**
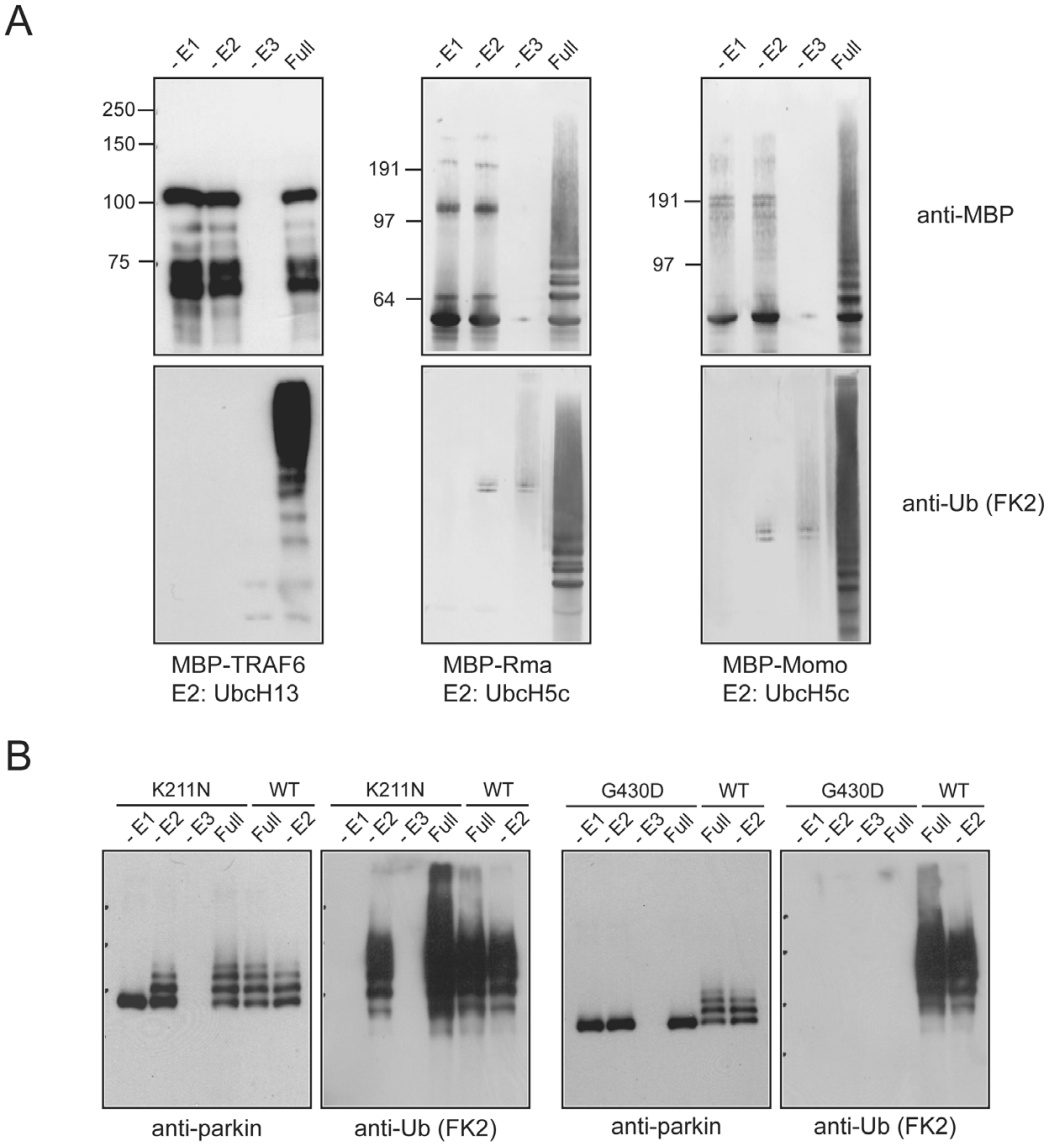
E2-independent activity is specific to parkin and is dependent on RING2 integrity. (A) *In vitro* ubiquitination reaction products generated by MBP-TRAF6, Rma or Momo in the absence of E1, E2 or E3, or in the presence of all three components including their respective cognate E2 (Full) were subjected to immunoblotting with anti-MBP and anti-FK2, as indicated. Unlike parkin, none of these E3 ligase could catalyze E2-independent ubiquitination. (B) Reaction products generated by disease-associated MBP-parkin K211N and G430D mutants in the absence of E1, E2 or E3, or in the presence of all three components (Full) were subjected to immunoblotting with anti-parkin and anti-FK2, as indicated. Reaction products catalyzed by wild type MBP-parkin in the presence or absence of UbcH7 were immunoblotted alongside for comparison.

To address the specificity of parkin-mediated E2-independent ubiquitination, we tested a spectrum of E3 ligases including TRAF6, Momo and Rma1, as well as RING finger domains of Trim5, Trim32, Rbx2, March8, Rnf4 and Deltex2, and found that none of these enzymes exhibit appreciable activity in the absence of their respective cognate E2s (Fig. 2A and S2A). On the other hand, all the E3s examined mediate robust self-ubiquitination in full reaction mixtures containing their cognate E2s (Fig. 2A and S2A). Thus, parkin appears uniquely endowed with E2-independent activity. However, as we have observed earlier, neither this activity nor the one produced in collaboration with UbcH7 is evident in the C441R mutant (Fig. 1A). Notably, we and others have previously demonstrated that RING2 mutations abolish parkin-mediated catalysis, whereas several parkin mutants harboring mutations outside of RING2 are catalytically-competent [9], [11]. Accordingly, we examined the ability of several disease-associated parkin mutants, including K211N, T240R, T415N and G430D, to mediate E2-independent ubiquitination. Consistent with previous reports [9], [11], we found that RING2 parkin mutants remain catalytically-null both in the presence or absence of E2s while mutations outside of parkin's RING2 retain their E2-dependent as well as E2-independent catalytic competency (Fig. 2B and S2B). Thus, the presence of an intact RING2 is essential for both E2-dependent and E2-independent parkin-mediated ubiquitination.

### MS analysis reveals different ubiquitin topologies associated with MBP-parkin and IBR-R2-catalyzed reactions in the absence or presence of E2

**Figure 3:**
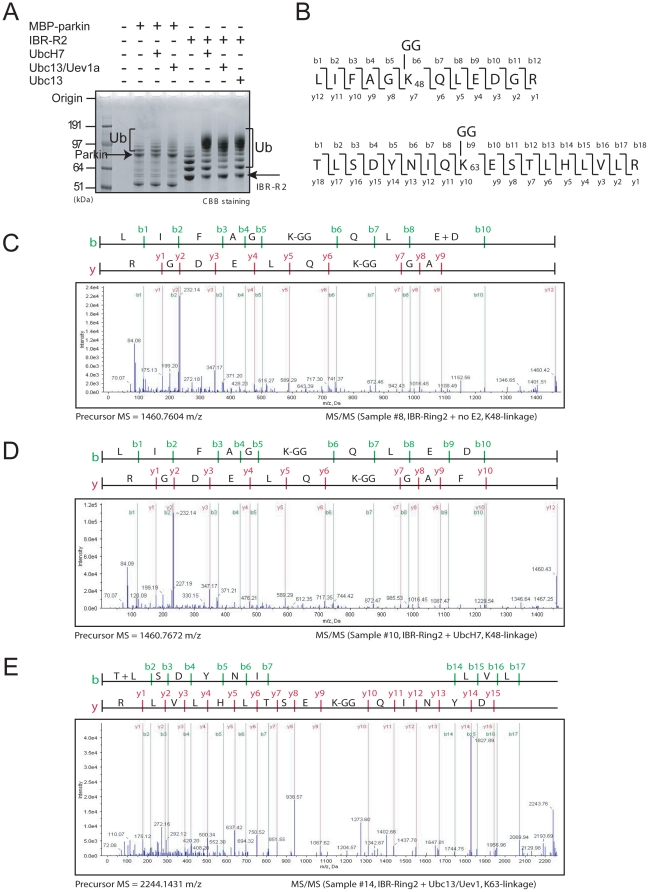
MS analysis of reaction products catalyzed by MBP-parkin or IBR-R2. (A) CBB-stained gel showing the reaction products produced by MBP-parkin or IBR-R2 under different conditions, as indicated. (B) Schematic diagrams of b and y ion assignments of K48- and K63-linkage ubiquitin peptides (C-E) MS/MS analysis of K48- or K63-linked ubiquitin chains catalyzed by IBR-R2. Precursor peaks estimated as K48- and K63-linkages were subsequently confirmed by MS/MS analysis.

To examine the nature of E2-independent and E2-dependent ubiquitination mediated by MBP-parkin and IBR-R2, we analyzed the reaction products directly via MS (Fig. 3). Although we detected ubiquitin via MS in MBP-parkin-catalyzed reactions both in the presence or absence of UbcH7, there is no evidence of isopeptide linkages indicative of ubiquitin chain assemblies (Table 1), suggesting that ubiquitin molecules detected are in their monomeric form and that MBP-parkin catalyzes solely monoubiquitination. This result is consistent with our earlier observations as well as previous reports demonstrating that parkin mediates monoubiquitination *in vitro* [9], [11]. In contrast, we detected the presence of both monoubiquitin as well as polyubiquitin linked specifically via K48 in reactions containing IBR-R2 in the presence or absence of UbcH7 (Fig. 3C–D and Table 1). In view of this and previous results by several groups showing that parkin could also collaborate with Ubc13/Uev1a to mediate K63-linked polyubiquitination [8], [10], [12], we wondered whether IBR-R2-catalyzed ubiquitin chain assembly could be modified by the heterodimeric E2 pair. Consistent with this, MS analysis of reaction products catalyzed by IBR-R2 in the presence of Ubc13/Uev1a reveals the presence of both K48 as well as K63-linked polyubiquitin species, along with monoubiquitin (Fig. 3E, S3–6 and Table 1). On the other hand, neither K48 nor K63-linked ubiquitin chains are detected when IBR-R2 is absent or is replaced by MBP-parkin (Fig. S4–6 and Table 1). Taken together, our results suggest that IBR-R2 contains an intrinsic activity that catalyzes polyubiquitin chains and that this activity is masked in full length parkin, which catalyzes solely monoubiquitination *in vitro*.

**Table 1:** Summary of MS/MS results.

	E2	Ubiquitin Linkage Topology
Parkin (full length)	no E2	no-linkage
	UbcH7	no-linkage
	Ubc13/Uev1	no-linkage
IBR-Ring2	no E2	K48
	UbcH7	K48
	Ubc13/Uev1	K48, K63
Δ152	no E2	no-linkage
	UbcH7	no-linkage
Δ237	no E2	K48

### Linker region represses intrinsic polyubiquitination activity of parkin

**Figure 4:**
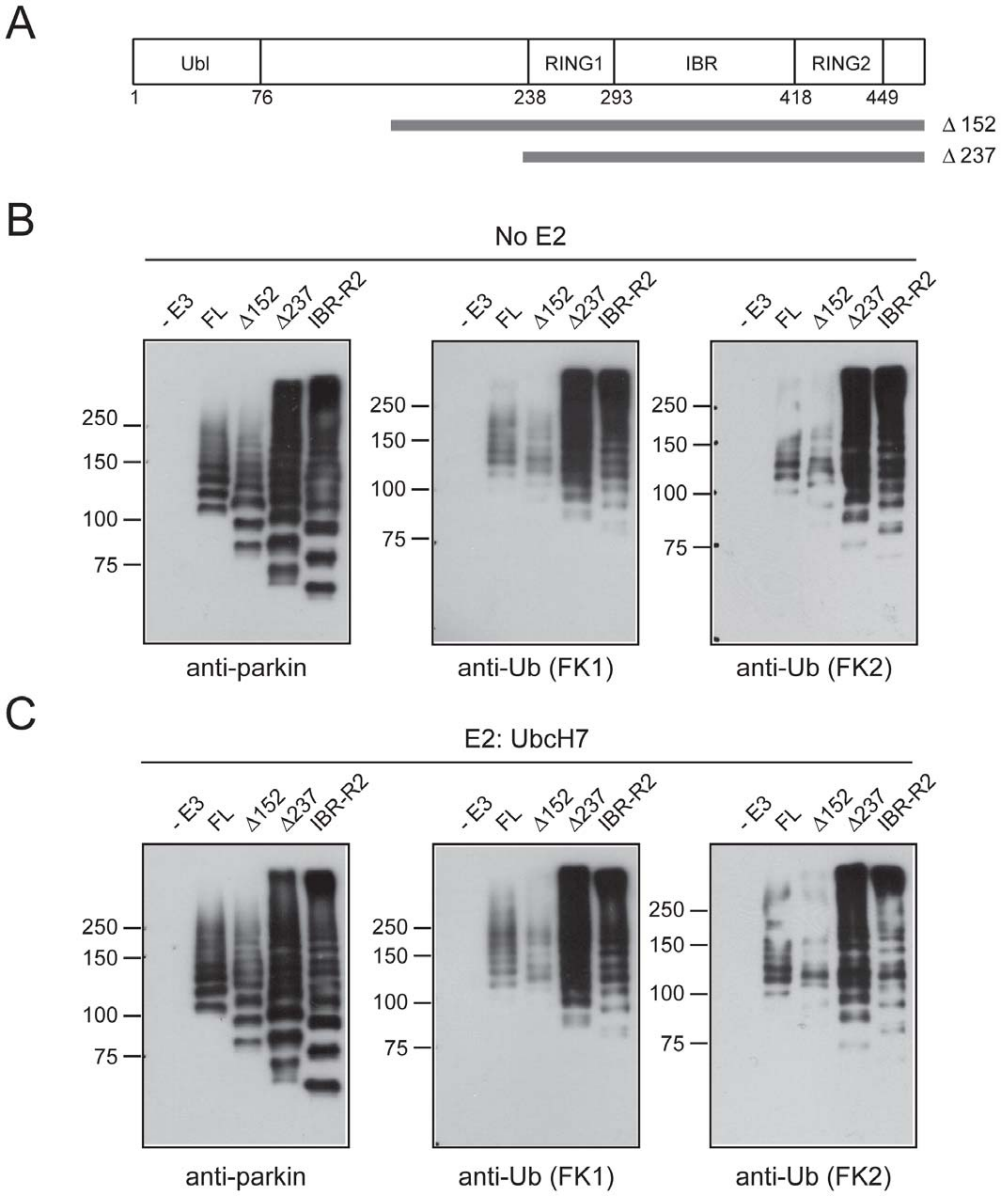
N-terminal region of parkin represses its intrinsic polyubquitination activity. (A) Schematic depiction of full length parkin protein and the deletion mutants Δ152 and Δ237. (B) *In vitro* ubiquitination reaction products generated by MBP-parkin, Δ152, Δ237 or IBR-R2 in the absence of E2 were subjected to immunoblotting with anti-parkin, anti-FK1 and anti-FK2, as indicated. (C) As in (B) except that reactions were conducted in the presence of UbcH7.

Given that IBR-R2 is devoid of parkin's N-terminal sequence, it is conceivable that the N-terminal region of parkin may repress its intrinsic polyubiquitination activity as exhibited by IBR-R2. To examine this possibility, we generated several parkin mutants with various lengths of their N-terminal sequence deleted (Fig. 4A and S7A) and assayed their activities. A truncated parkin mutant that is deleted of its entire Ubl domain (ΔUbl) behaves essentially like the wild type protein (Fig. S7B), as are mutants that are partially devoid of their linker region (Fig. 4B and S7B). However, a parkin mutant (Δ237) containing RING1 and IBR-R2 sequences but is completely deleted of its Ubl and linker region sequences exhibits catalytic properties that bear striking resemblance to that displayed by IBR-R2 (Fig. 4B). Notably, the Δ237 mutant generated robust FK1-immunoreactive species both in the absence and presence of E2 (Fig. 4B), suggesting a capacity of the mutant to mediate polyubiquitination. In contrast, a corresponding deletion mutant (Δ152) that retained a portion of the linker region from amino acid 152–237 fails to generate appreciable FK1-immunoreactive products (Fig. 4B). Supporting this, MS analysis of reaction products catalyzed by Δ237 mutant in the absence or presence of E2 (UbcH7) reveals the presence of K48-linked ubiquitin chains, whereas no isopeptide linkages are detectable in reaction products catalyzed by the Δ152 parkin mutant (Table 1 and Fig. S8). Taken together, our results suggest that the stretch of parkin sequence at the linker region from amino acid 152–237 likely exerts repression on its intrinsic polyubiquitination activity.

**Figure 5:**
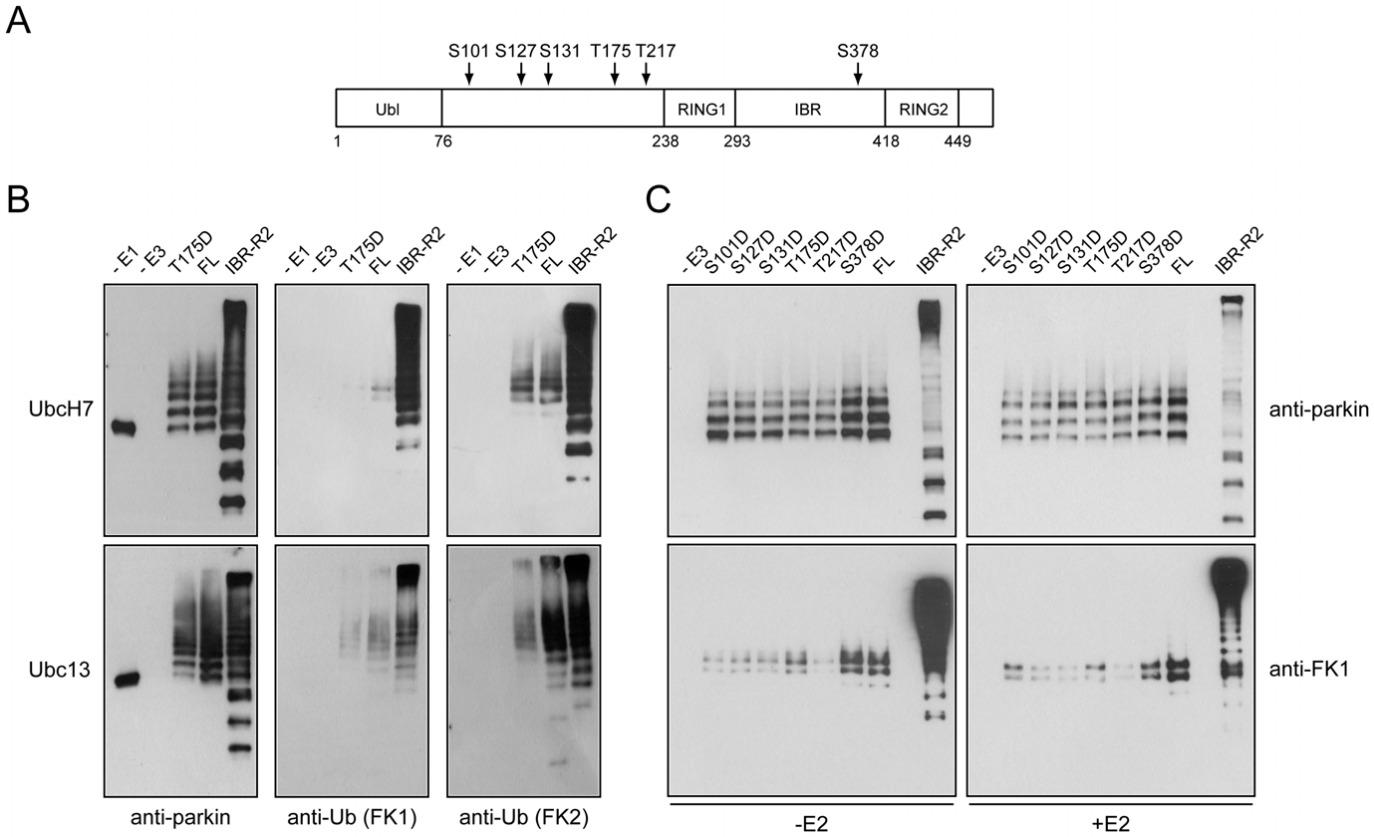
Parkin S/T-D phospho-mimetic mutants display similar ubiquitination profile as the wild type protein. (A) Schematic depiction of full length parkin protein with the position of known S/T phosphorylation sites indicated by arrows (B) *In vitro* ubiquitination reaction products generated by MBP-parkin, MBP-parkin T175D or IBR-R2 in the presence of UbcH7 or Ubc13 were subjected to immunoblotting with anti-parkin, anti-FK1 and anti-FK2, as indicated. (C) *In vitro* ubiquitination reaction products generated by various parkin phospomimetic mutants in the absence or presence of E2 (UbcH7) were subjected to immunoblotting with anti-parkin and anti-FK1.

Notwithstanding the above, how the intrinsic polyubiquitination activity of parkin becomes unmasked in the full length protein is unclear. However, a recent study by Sha and colleagues has demonstrated that parkin phosphorylation by PINK1 activates its poly-ubiquitination activity, although the exact site(s) where parkin is phosphorylated was not mapped [13]. In a related study, Kim and colleagues found that threonine-175 (T175) on parkin is a major residue phosphorylated by PINK1 [15]. Notably, T175 resides within the “repressor” sequence (i.e. a.a. 152–237) of parkin that we have identified above and it is attractive to speculate that its modification via phosphorylation might stimulate the intrinsic polyubiquitination activity of the enzyme. To examine this possibility, we generated MBP-parkin T175D phospho-mimetic mutant and assayed its activity alongside full length parkin and IBR-R2. Additionally, we also generated various other parkin phospho-mimetic mutants including S101D, S127D, S131D, T217D and S378D (Fig. 5A). All of these residues on parkin have been reported to undergo phosphorylation *in vivo* [15], [16], [17], [18]. However, we found that MBP-parkin T175D behaves catalytically like the wild type full length protein in the absence or presence of UbcH7 (Fig. 5B & C). Further, switching the E2 from UbcH7 to Ubc13 did not alter the ubiquitination profile of the mutant relative to wild type parkin (Fig. 5B). Similar observations were also made with other phospho-mimetic mutants examined (Fig. 5C). Thus, mimicking parkin phosphorylation via S-D substitution does not appear to result in the unmasking of the enzyme's intrinsic polyubiquitination activity.

### IBR-R2 but not full length parkin promotes polyubiquitination of synphilin-1

**Figure 6:**
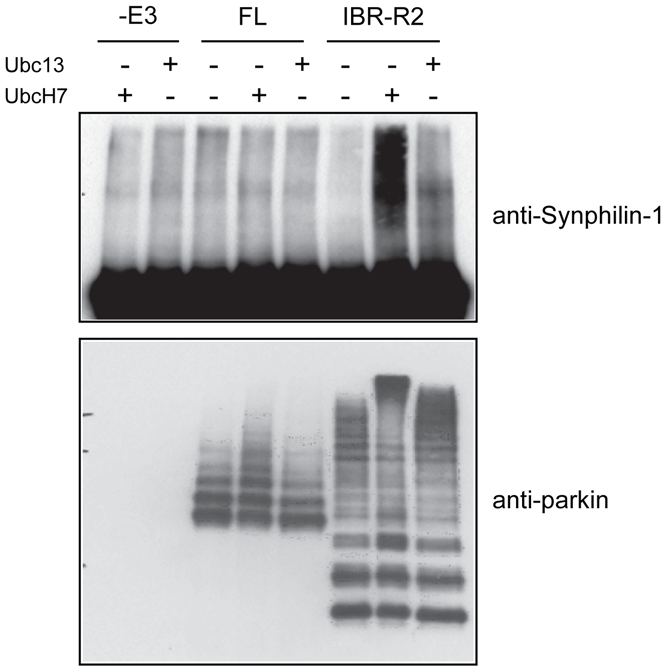
IBR-R2 but not full length parkin promotes synphilin-1ubiquitination. *In vitro* ubiquitination reaction was carried out with synphilin-1 as a substrate in the absence of parkin (i.e. –E3) or in the presence of MBP-parkin (FL) or IBR-R2 in combination with either UbcH7, Ubc13 or no E2 (as indicated). Parkin-mediated ubiquitination of synphilin-1 was analyzed by means of anti-synphilin-1 immunoblotting (*top panel*) whereas parkin-mediated self ubiquitination was analyzed by means of anti-parkin immunoblotting (*bottom panel*).

Notably, our above results regarding parkin's catalytic properties were derived from its auto-ubiquitination activity. We were therefore curious to examine how full length parkin and IBR-R2 might ubiquitinate a physiological substrate in the presence or absence of E2. For this purpose, we have prepared recombinant synphilin-1 (an α-synuclein interactor that we have previously demonstrated to be a substrate of parkin [10]) and subjected the protein to parkin-mediated ubiquitination *in vitro*. Anti-synphilin-1 immunoblotting, which allows us to assess synphilin-1 ubiquitination directly (against the background of parkin auto-ubiquitination), revealed that both full length parkin and IBR-R2 fail to promote synphilin-1 ubiquitination in the absence of E2 (Fig. 6). However, in the presence of UbcH7, parkin IBR-R2 but not the full length protein mediates robust synphilin-1 ubiquitination, an observation that is consistent with the ability of IBR-R2 to catalyze polyubiquitination (Fig. 6). The auto-ubiquitination activity of parkin in the absence or presence of E2 is otherwise not appreciably affected by synphilin-1 (Fig. 6). Curiously, IBR-R2-mediated synphilin-1 ubiquitination is considerably weaker when UbcH7 is replaced by Ubc13/Uev1a (Fig. 6), suggesting that additional factors/events present in cellular system might account for parkin-mediated K63-linked ubiquitination of synphilin-1 that we have previously observed *in vivo* [10].

## Discussion

We have demonstrated here an unprecedented ability of an E3 member to mediate E2-independent ubiquitin modification. This is intriguing, as it markedly differs from the canonical mechanism of protein ubiquitination where sequential actions of E1, E2 and E3 are needed to catalyze the addition of ubiquitin molecules on a substrate. Further, in the “sequential” model, it is widely presumed that following the initial identification and conjugation of a substrate with ubiquitin, the same E2/E3 complex is essential for the assembly of polyubiquitin chain on the substrate [19], [20], [21]. Notably, none to date has established that E3 in the absence of E2 is capable of catalyzing protein ubiquitination, although one group has recently demonstrated that ubiquitin chain can be preassembled on an E2 before being transferred to a substrate by an E3 [22], whereas another has shown that E1 is capable of extending polyubiquitin chain on E2 [23]. Here, we have established that the ubiquitin ligase activity of parkin can support ubiquitination (albeit auto-ubiquitination) *in vitro* in the complete absence of E2. Further, we have demonstrated that parkin-mediated E2-independent ubiquitination is a rather specific property of the enzyme, as several other related E3 ligases that we have tested alongside are clearly devoid of this activity. Taken together, parkin thus appears to be a unique ubiquitin ligase capable of recruiting E1 directly (i.e. without an intermediate E2 member) to carry out protein ubiquitination.

Notwithstanding the above, there are a few caveats in our findings that are worth highlighting. Firstly, we do not know at this moment whether and how the proposed transfer of activated ubiquitin from E1 to parkin occurs, i.e. whether the transfer occur through the internal E1-ubiquitin thioester of the ternary complex or through the bound ubiquitin adenylate intermediate and whether it involves the active site cysteine of parkin. Secondly, our *in vitro* assays (as with the case with the majority of those reported in the literature) were conducted in the presence of the reducing agent DTT, which can act as an acceptor of E1 ubiquitin thioester in the absence of E2. In this case, parkin might be capable of orienting ubiquitin-DTT moieties on its surface to catalyze by proximity effect a slow basal rate of ubiquitination. However, as reducing agents are required to prevent cysteine modifications and as such to maintain the integrity of RING domains, it is difficult to evaluate the *in vitro* activity of parkin(or other RING-containing E3 ligases) in the absence of DTT. Importantly, our results demonstrated that the rate of parkin-mediated E2-independent reaction, as shown in Fig. 1C, is not exactly sluggish, as appreciable product formation could be detected shortly after the start of reaction. Furthermore, a mechanism based on proximal effect would not provide an adequate explanation to the different modes of ubiquitination (i.e. mono- and polyubiquitination) exhibited by full length parkin and IBR-R2 in the apparent absence of E2 (notwithstanding that no other E3 examined in this study is capable of E2-independent ubiquitination).

Importantly, we also showed that parkin contains an intrinsic polyubiquitination activity that is normally masked in the full length protein. As a result, full length parkin catalyzes solely monoubiquitination *in vitro* regardless of the presence or absence of E2, a phenomenon that is consistent with previous reports by ourselves and others [9], [11]. Surprisingly, full length parkin-catalyzed reactions exhibit some immunoreactivity towards FK1 antibody (albeit modestly so relative to IBR-R2-catalyzed reactions), which specifically recognizes polyubiquitin chains. It is possible that anti-FK1 may exhibit a low level of cross-reactivity with multiple-monoubiquitinated products, which parkin is known to mediate [9], [11]. When unmasked, the intrinsic activity encoded within the sequence of IBR-R2 supports both E2-independent and E2-dependent assembly of ubiquitin chains. However, the E2-independent activity applies to parkin self-ubiquitination and not to its activity towards its substrate (at least with synphilin-1). Indeed, the effect of E2 on parkin-mediated ubiquitination becomes significantly more pronounced when the enzyme is acting on its substrate. Consistent with the repressed state of the full length protein, only IBR-R2 is capable of mediating appreciable synphilin-1 ubiquitination in the presence of E2. It is currently unclear how the intrinsic polyubiquitination activity of parkin becomes activated in the full length protein. Although none of our single phospho-mimetic parkin mutants seems to work, it is possible that the activation event requires multiple parkin phosphorylation, or perhaps the recruitment of an unknown activator by the enzyme. Notably, several recent reports from our laboratories and others have uncovered parkin's function in the surveillance pathway for damaged mitochondria [24], [25], [26], [27], [28], [29], [30]. During this process, parkin specifically polyubiquitinates depolarized mitochondrial proteins. Interestingly, the ubiquitin ligase activity of parkin is repressed in the cytoplasm under steady-state conditions in cells; however, PINK1-dependent mitochondrial localization liberates the latent enzymatic activity of Parkin [28], whereupon it promotes the degradation of mitochondrial outer membrane proteins [31], [32]. We speculate that the full-length parkin whose polyubiquitination activity is repressed *in vitro* may reflect inactivated parkin in the cytoplasm under steady state conditions in cells. Further, consistent with our findings described here, SILAC analysis revealed a substantial increase in parkin-mediated K48-linked (9-fold) and K63-linked (28-fold) polyubiquitination upon mitochondrial depolarization [33].

It is noteworthy that our MS data reveal that IBR-R2 catalyzes the formation of K48-linked ubiquitin chain in an E2-independent manner, suggesting that once unmasked, parkin-mediated polyubiquitination is inherently primed for proteasome degradation. Alternatively, when Ubc13/Uev1a is recruited, parkin could modify its substrate (or itself) via K63-linked ubiquitin chains, although our results with recombinant synphilin-1 would suggest the involvement of additional factor/events for parkin-mediated K63 ubiquitination to occur *in vivo*. The multiple modes of parkin-mediated ubiquitination would presumably endow the protein with a greater flexibility to react to changing cellular conditions. For example, upon mitochondrial depolarization, parkin is known to modify proteins on the damaged organelles via a variety of ubiquitin linkages, including K27, K48 and/or K63 [24], [29]. Further, in times of proteasomal stress, parkin-mediated K63 ubiquitination may be favored over the proteasome-linked K48 ubiquitination as the former mode of ubiquitin modification has the ability to divert protein load away from an otherwise overwhelmed proteasome (reviewed in [34]) as well as enhance the pro-survival NFκB pathway [35].

In conclusion, we have provided evidence here demonstrating that parkin contains an intrinsic polyubiquitination activity that is normally masked in the full length state. We also showed that parkin is a unique E3 member not only in terms of its ability to mediate multiple forms of E2-dependent ubiquitination, but also its ability to mediate ubiquitination in the absence of E2. However, we remain cognizant of the several caveats associated with our current findings, which we hope would be clarified by future studies.

## Supporting Information

Figure S1 **MBP-parkin catalyzes E2-independent ubiquitination.** (A) *Left*, Schematic depiction of various recombinant MBP-parkin proteins including MBP-parkin (FL), MBP-parkin C441R (C441R) and MBP-parkin IBR-R2 (IBR-R2). *Right*, Coomassie Brillant Blue (CBB)-stained gel and anti-parkin immunoblots showing the purity of the various recombinant parkin species. (B & C) *In vitro* ubiquitination reaction products generated by MBP-parkin in the presence or absence of UbcH7 and different forms of E1 were subjected to immunoblotting with anti-parkin and anti-FK2, as indicated.
(PDF)

Figure S2 **Other E3 members as well as parkin RING2 mutants are devoid of E2-independent activity.** *In vitro* ubiquitination reaction products generated by purified MBP-proteins containing the catalytic RING domain of various E3 members in the presence or absence of their cognate E2, UbcH5, were subjected to immunoblotting with anti-FK2, as indicated. (B) Reaction products generated by MBP-parkin T240R and T415N (RING2 domain) mutant in the absence of E1, E2 or E3, or in the presence of all three components (Full) were subjected to immunoblotting with anti-parkin. Reaction products catalyzed by wild type MBP-parkin in the presence of UbcH7 were immunoblotted alongside for comparison.
(PDF)

Figure S3 **Parkin-mediated ubiquitination in the presence of Ubc13/Uev1a.** *In vitro* ubiquitination reaction products generated by MBP-parkin or IBR-R2 in the presence or absence of Ubc13/Uev1a under different conditions were subjected to immunoblotting with anti-parkin, anti-FK1 and anti-FK2, as indicated.
(PDF)

Figure S4 **Sample collection for MS analysis.** (A–C) CBB-stained gel showing the reaction products produced by MBP-parkin or IBR-R2 under different conditions, as indicated. Portion of gels corresponding to ubiquitinated protein species used for MS analysis are shown alongside.
(PDF)

Figure S5 **K48-linked ubiquitin chains associated with IBR-R2 catalyzed reactions in the presence of Ubc13/Uev1a.** MS results derived from IBR-catalyzed reaction products in the presence of Ubc13/Uev1a revealing the presence of both K48-linked ubiquitin. The peak corresponding to K48–linkages is indicated.
(PDF)

Figure S6 **K63-linked ubiquitin chains associated with IBR-R2 catalyzed reactions in the presence of Ubc13/Uev1a.** Besides K48-linked ubiquitin chains, MS results derived from IBR-catalyzed reaction products in the presence of Ubc13/Uev1a also revealed the presence of K63-linked ubiquitin. The peak corresponding to K63-linkages is indicated.
(PDF)

Figure S7 **Lack of E2-independent activity in several parkin deletion mutants** (A) Schematic depiction of full length parkin protein, IBR-R2 and various deletion mutants (B) *In vitro* ubiquitination reaction products generated by the various MBP-parkin species in the absence of E2 were subjected to immunoblotting with anti-parkin and anti-FK1, as indicated.
(PDF)

Figure S8 **Parkin Δ237 mutant exhibits E2-independent and E2-dependent polyubiquitination activity.** (A) CBB-stained gel showing the reaction products produced by MBP-parkin Δ152 or Δ237 mutant under different conditions, as indicated. (B) CBB-stained gel showing excised portion of gels corresponding to ubiquitinated protein species produced by MBP-parkin Δ152 or Δ237 were used for MS analysis. (C–D) MS results derived from MBP-parkin Δ237-catalyzed reaction products revealing the presence of K48-linked ubiquitin.
(PDF)
