# Supplementary figures and images for: Parkin Mediates Apparent E2-Independent Monoubiquitination In Vitro and Contains an Intrinsic Activity That Catalyzes Polyubiquitination
Source: PLoS One. 2011 May 23;6(5):e19720. doi: 10.1371/journal.pone.0019720 (PMC3100294; doi:10.1371/journal.pone.0019720)

A

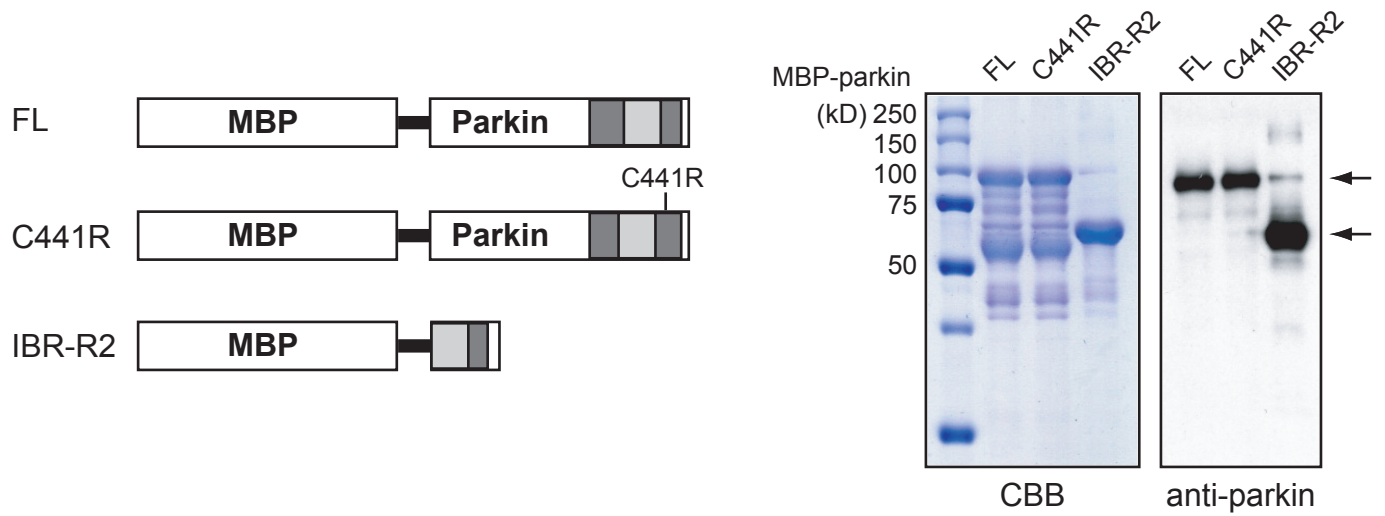

B

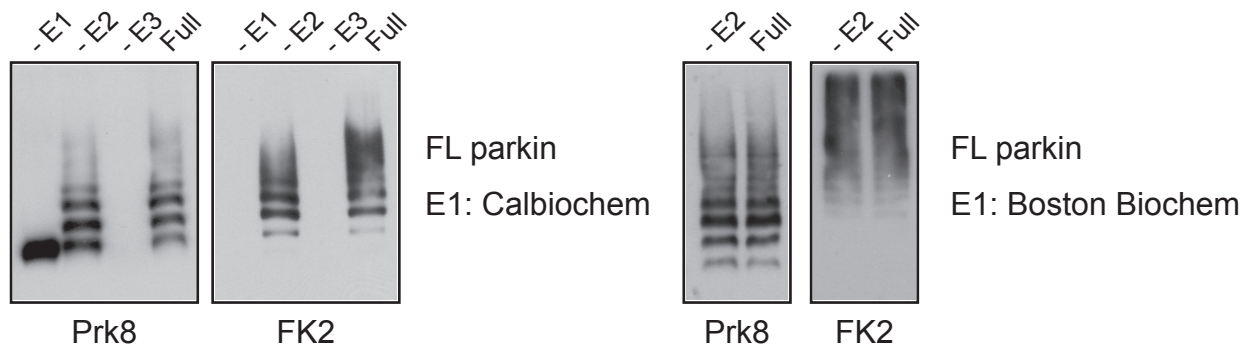

C

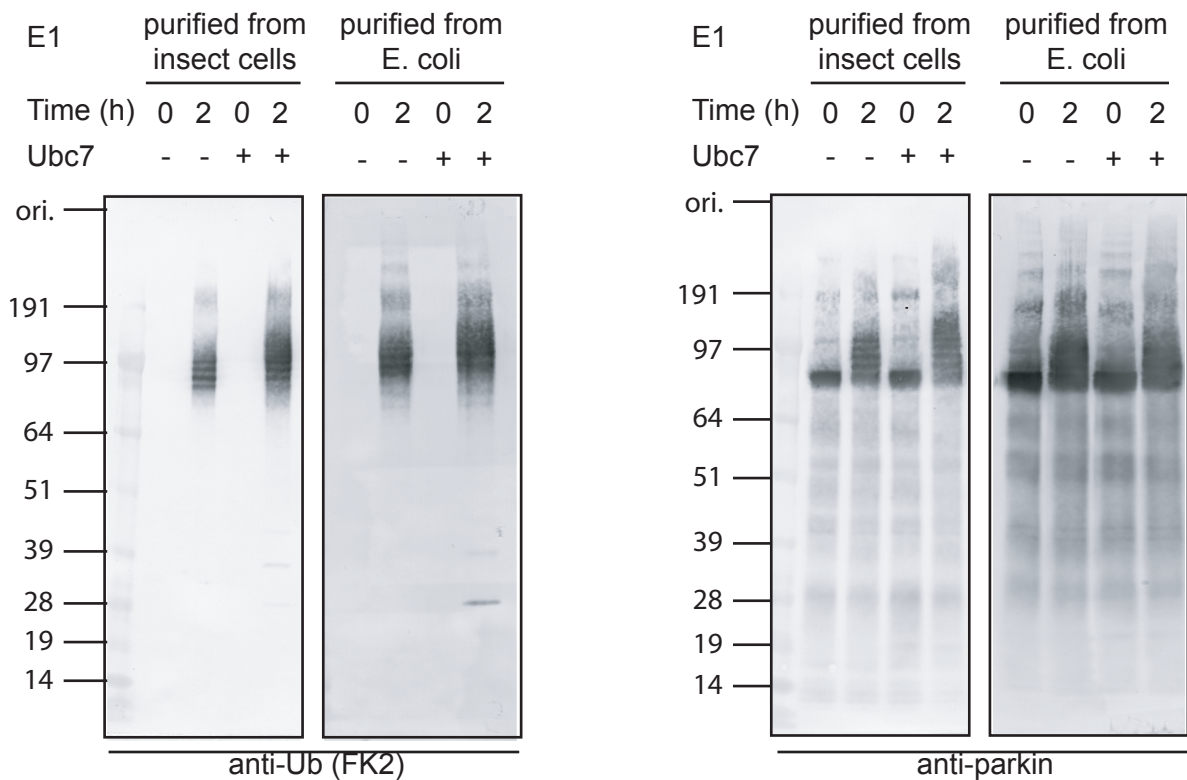

Supplement: Figure S1 — MBP-parkin catalyzes E2-independent ubiquitination. (A) Left, Schematic depiction of various recombinant MBP-parkin proteins including MBP-parkin (FL), MBP-parkin C441R (C441R) and MBP-parkin IBR-R2 (IBR-R2). Right, Coomassie Brillant Blue (CBB)-stained gel and anti-parkin immunoblots showing the purity of the various recombinant parkin species. (B & C) In vitro ubiquitination reaction products generated by MBP-parkin in the presence or absence of UbcH7 and different forms of E1 were subjected to immunoblotting with anti-parkin and anti-FK2, as indicated. (PDF) [file pone.0019720.s001.pdf]

Supplemental Fig. S2 Chew, Matsuda et al

A

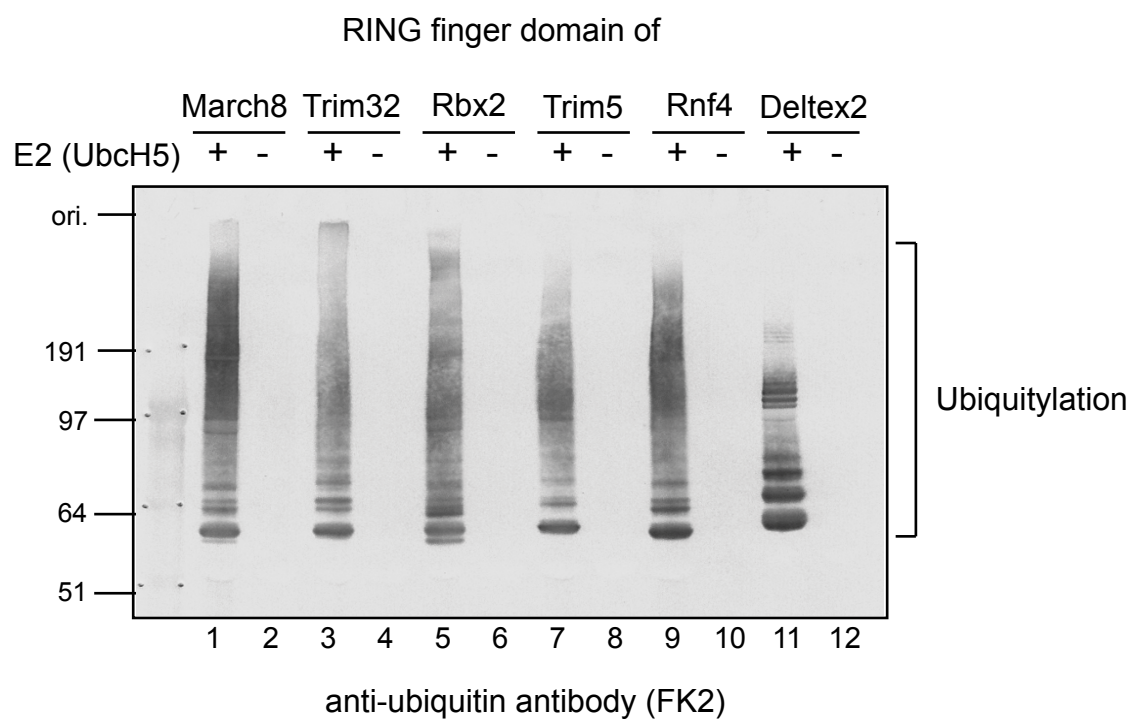

B

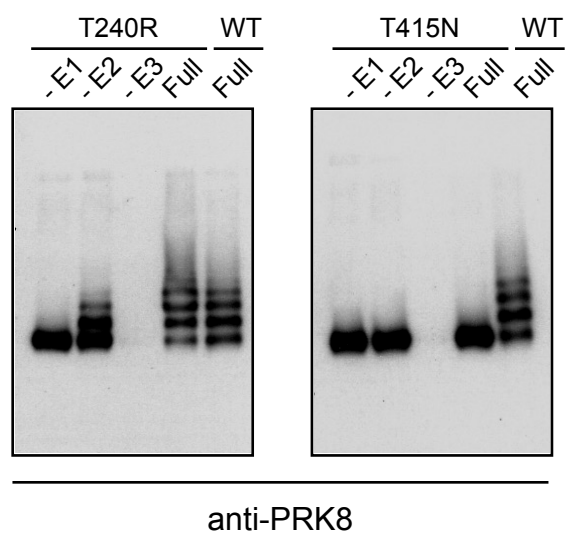

Supplement: Figure S2 — Other E3 members as well as parkin RING2 mutants are devoid of E2-independent activity. In vitro ubiquitination reaction products generated by purified MBP-proteins containing the catalytic RING domain of various E3 members in the presence or absence of their cognate E2, UbcH5, were subjected to immunoblotting with anti-FK2, as indicated. (B) Reaction products generated by MBP-parkin T240R and T415N (RING2 domain) mutant in the absence of E1, E2 or E3, or in the presence of all three components (Full) were subjected to immunoblotting with anti-parkin. Reaction products catalyzed by wild type MBP-parkin in the presence of UbcH7 were immunoblotted alongside for comparison. (PDF) [file pone.0019720.s002.pdf]

Supplemental Fig. S3 Chew, Matsuda et al

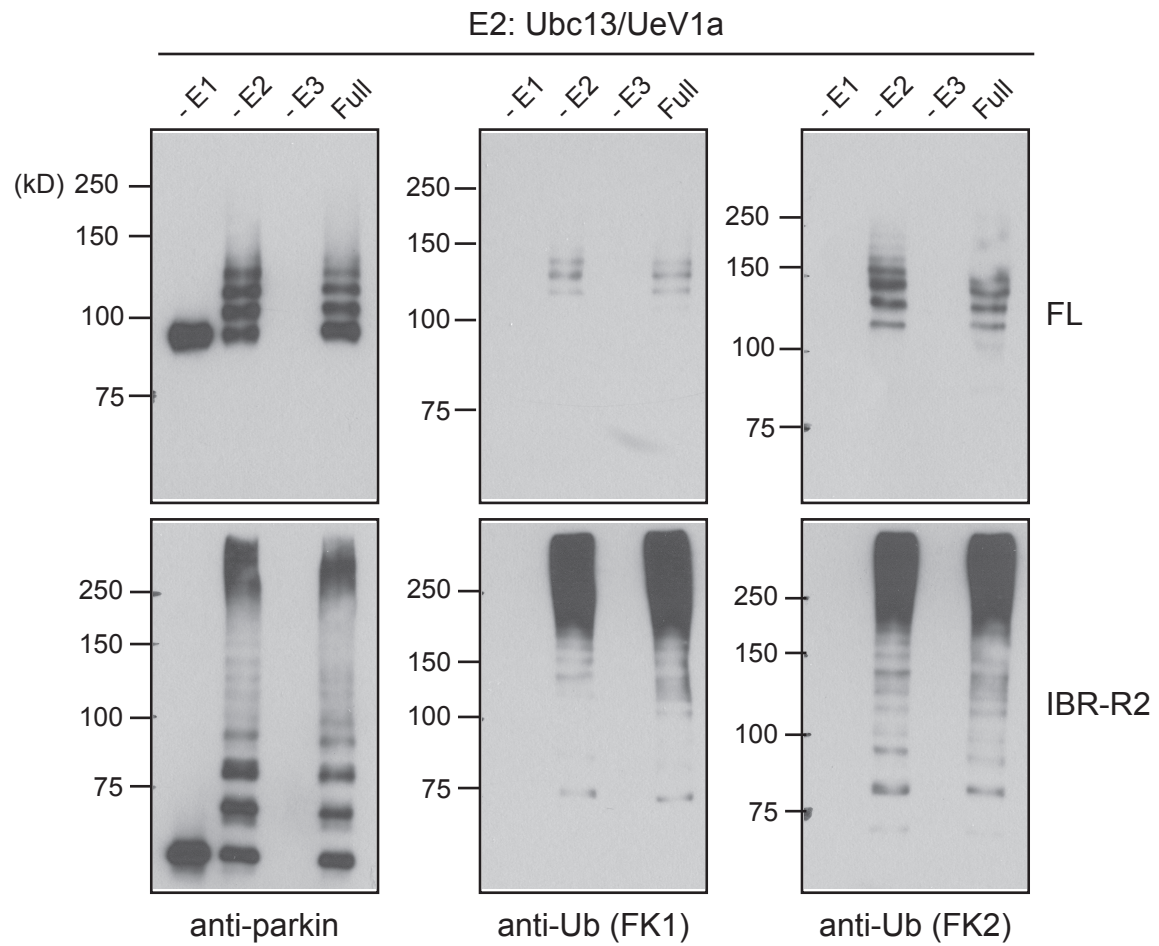

Supplement: Figure S3 — Parkin-mediated ubiquitination in the presence of Ubc13/Uev1a. In vitro ubiquitination reaction products generated by MBP-parkin or IBR-R2 in the presence or absence of Ubc13/Uev1a under different conditions were subjected to immunoblotting with anti-parkin, anti-FK1 and anti-FK2, as indicated. (PDF) [file pone.0019720.s003.pdf]

Supplemental Fig. S4 Chew, Matsuda et al

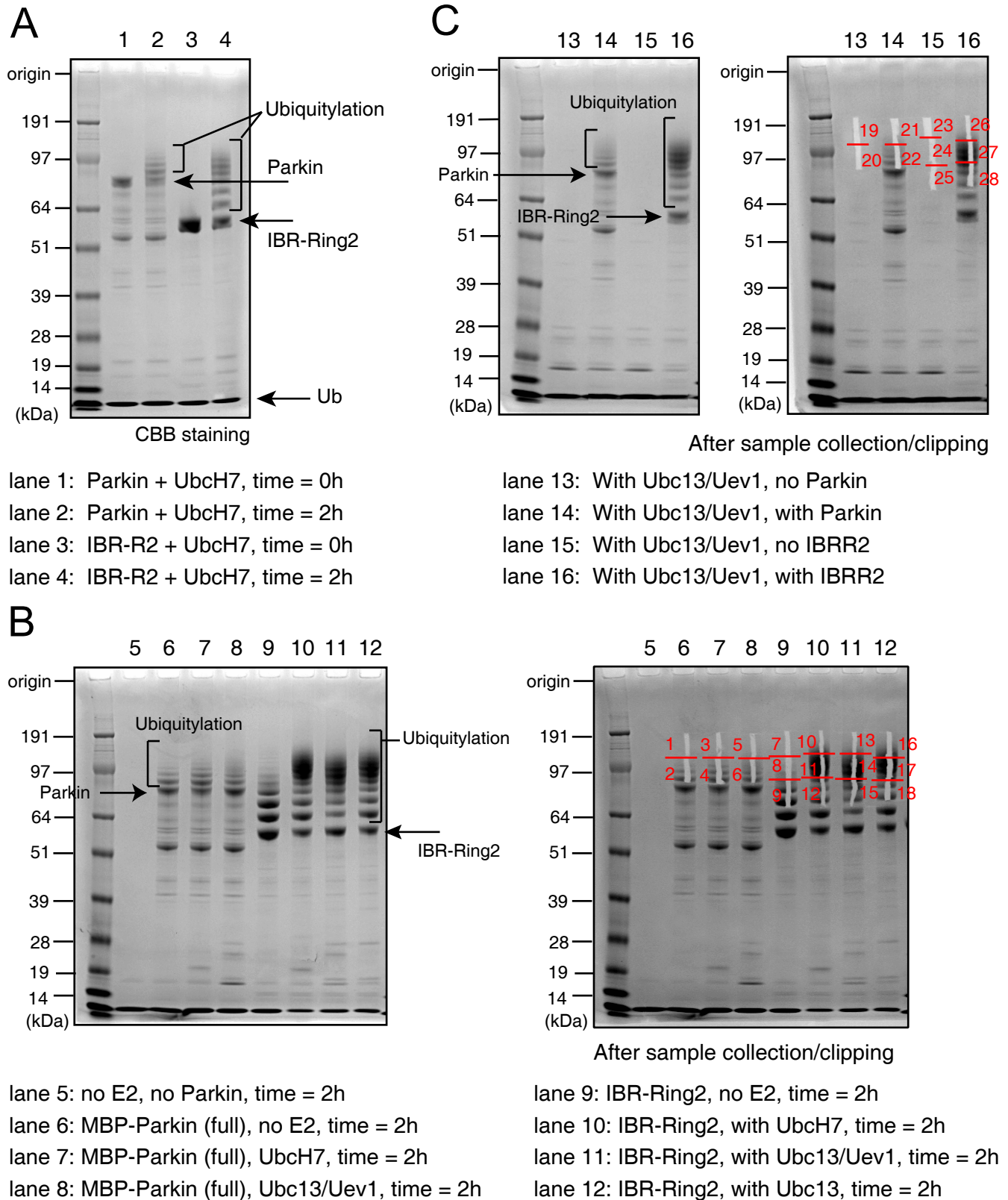

Supplement: Figure S4 — Sample collection for MS analysis. (A–C) CBB-stained gel showing the reaction products produced by MBP-parkin or IBR-R2 under different conditions, as indicated. Portion of gels corresponding to ubiquitinated protein species used for MS analysis are shown alongside. (PDF) [file pone.0019720.s004.pdf]

Supplemental Fig. S7 Chew, Matsuda et al

A

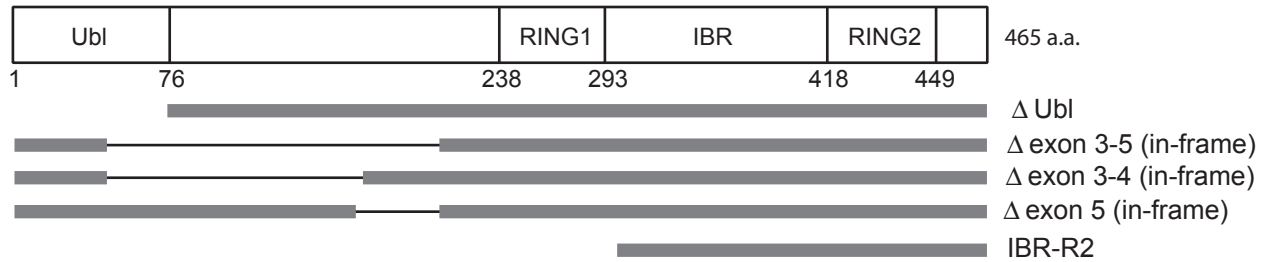

B

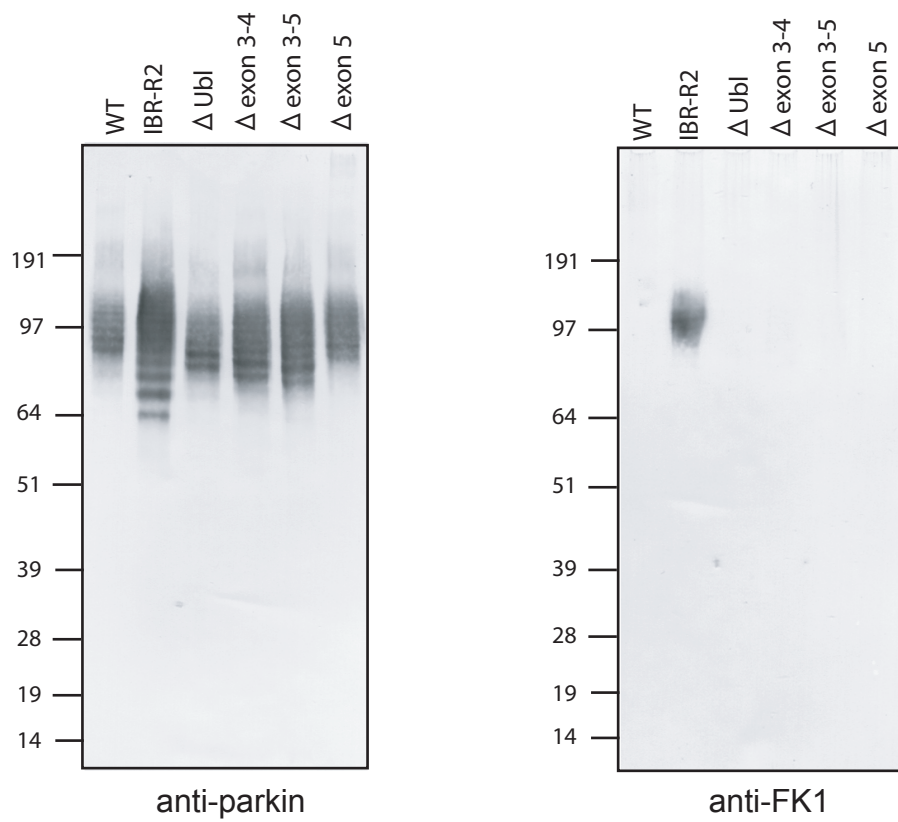

Supplement: Figure S7 — Lack of E2-independent activity in several parkin deletion mutants (A) Schematic depiction of full length parkin protein, IBR-R2 and various deletion mutants (B) In vitro ubiquitination reaction products generated by the various MBP-parkin species in the absence of E2 were subjected to immunoblotting with anti-parkin and anti-FK1, as indicated. (PDF) [file pone.0019720.s007.pdf]

# Supplemental Fig. S8 Chew, Matsuda et al

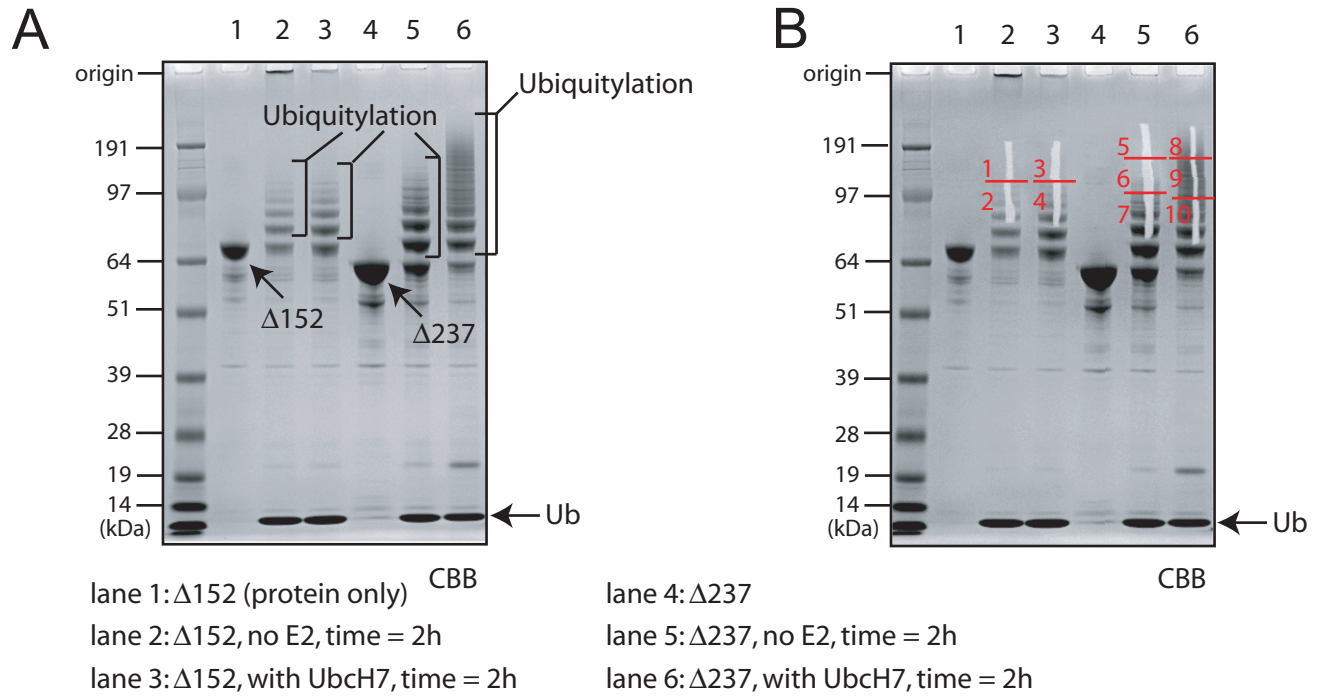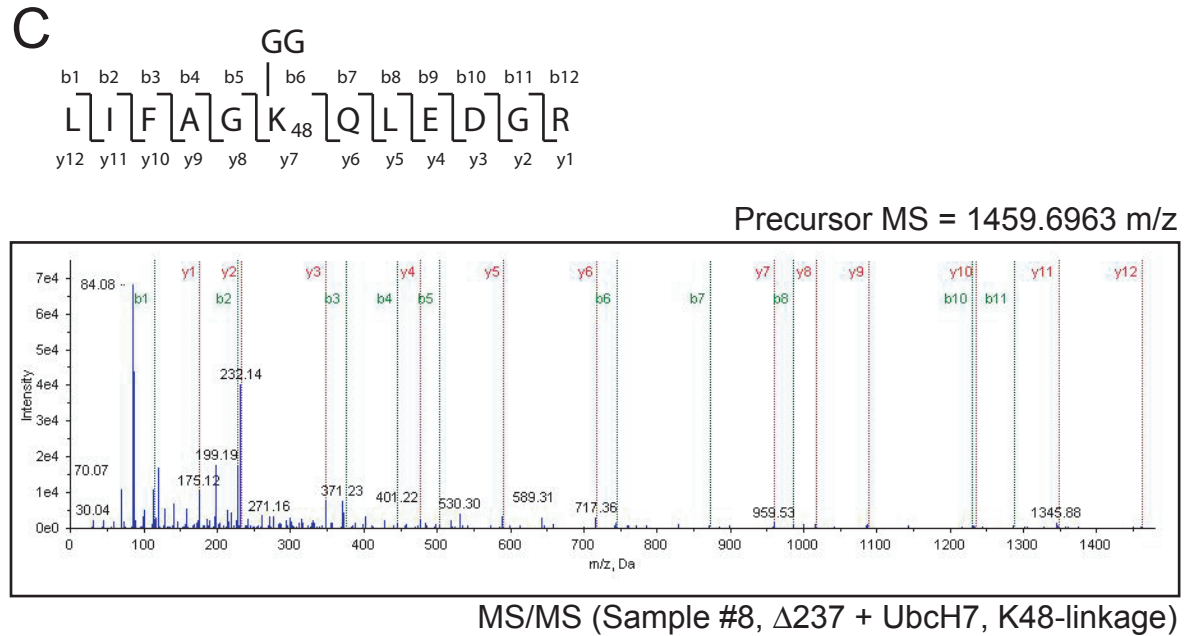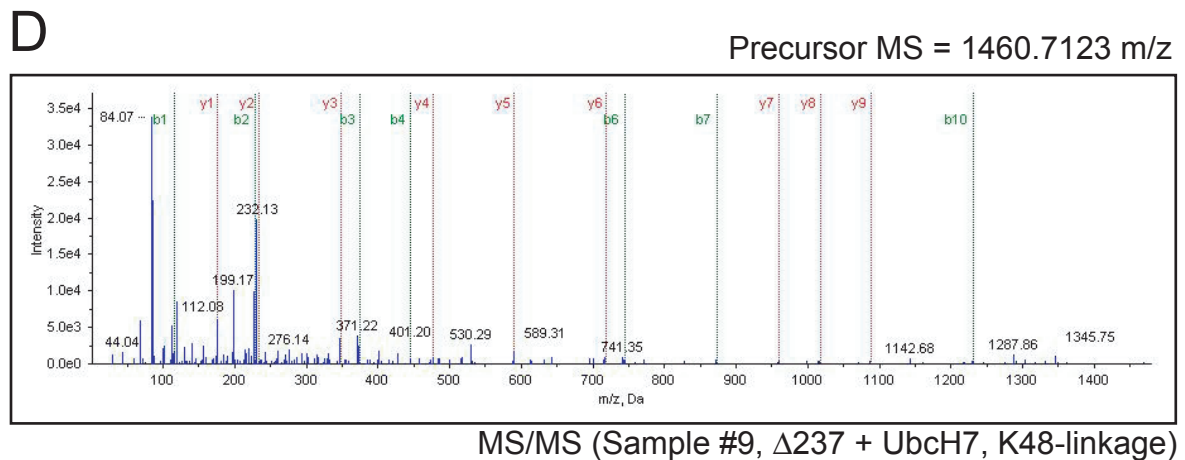

Supplement: Figure S8 — Parkin Δ237 mutant exhibits E2-independent and E2-dependent polyubiquitination activity. (A) CBB-stained gel showing the reaction products produced by MBP-parkin Δ152 or Δ237 mutant under different conditions, as indicated. (B) CBB-stained gel showing excised portion of gels corresponding to ubiquitinated protein species produced by MBP-parkin Δ152 or Δ237 were used for MS analysis. (C–D) MS results derived from MBP-parkin Δ237-catalyzed reaction products revealing the presence of K48-linked ubiquitin. (PDF) [file pone.0019720.s008.pdf]
