# Supplementary material for: Parkin Mediates Apparent E2-Independent Monoubiquitination In Vitro and Contains an Intrinsic Activity That Catalyzes Polyubiquitination
Source: PLoS One. 2011 May 23;6(5):e19720. doi: 10.1371/journal.pone.0019720 (PMC3100294; doi:10.1371/journal.pone.0019720)
Supplement: Figure S5 — K48-linked ubiquitin chains associated with IBR-R2 catalyzed reactions in the presence of Ubc13/Uev1a. MS results derived from IBR-catalyzed reaction products in the presence of Ubc13/Uev1a revealing the presence of both K48-linked ubiquitin. The peak corresponding to K48–linkages is indicated. (PDF) [file pone.0019720.s005.pdf]

Supplemental Fig. S5 Chew, Matsuda et al

A

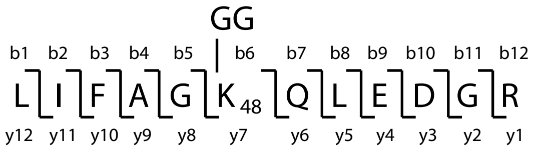

B

Precursor MS = 1460.7645 m/z

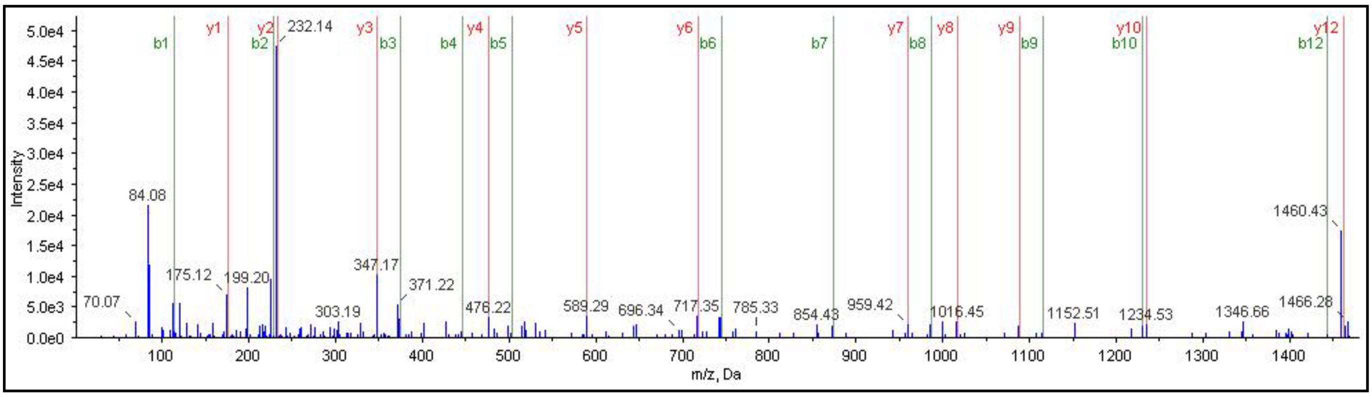

MS/MS (Sample #13, IBR-Ring2 + Ubc13/Uev1, K48-linkage)

C

Precursor MS = 1460.7610 m/z

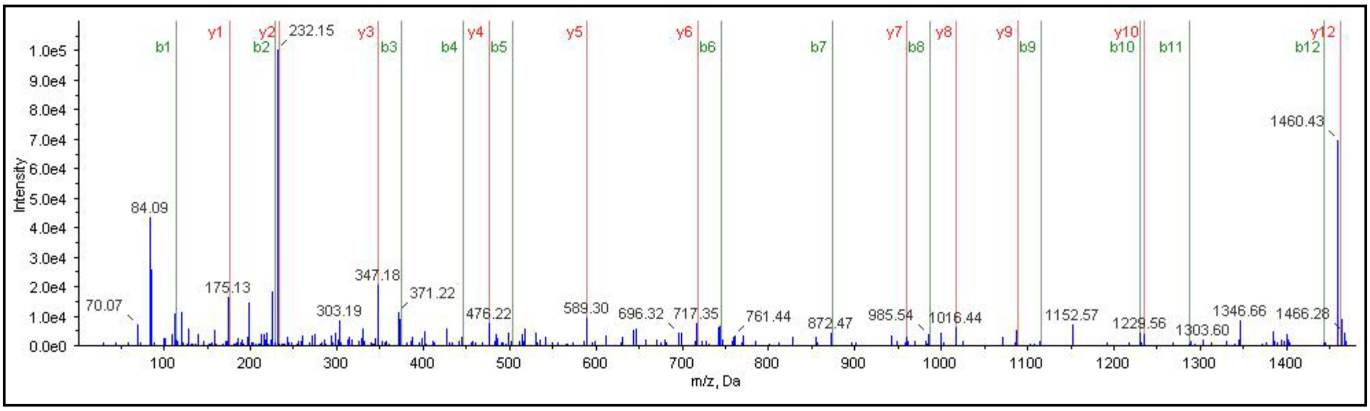

MS/MS (Sample #14, IBR-Ring2 + Ubc13/Uev1, K48-linkage)

D

Precursor MS = 1460.7562 m/z

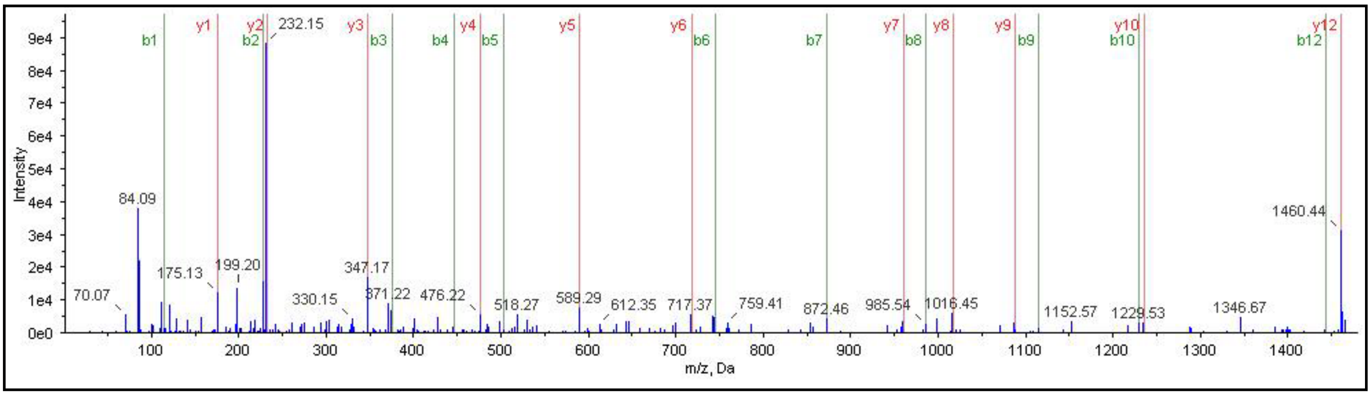

MS/MS (Sample #16, IBR-Ring2 + Ubc13, K48-linkage)
