# Supplementary material for: Parkin Mediates Apparent E2-Independent Monoubiquitination In Vitro and Contains an Intrinsic Activity That Catalyzes Polyubiquitination
Source: PLoS One. 2011 May 23;6(5):e19720. doi: 10.1371/journal.pone.0019720 (PMC3100294; doi:10.1371/journal.pone.0019720)
Supplement: Figure S6 — K63-linked ubiquitin chains associated with IBR-R2 catalyzed reactions in the presence of Ubc13/Uev1a. Besides K48-linked ubiquitin chains, MS results derived from IBR-catalyzed reaction products in the presence of Ubc13/Uev1a also revealed the presence of K63-linked ubiquitin. The peak corresponding to K63-linkages is indicated. (PDF) [file pone.0019720.s006.pdf]

Supplemental Fig. S6 Chew, Matsuda et al

A

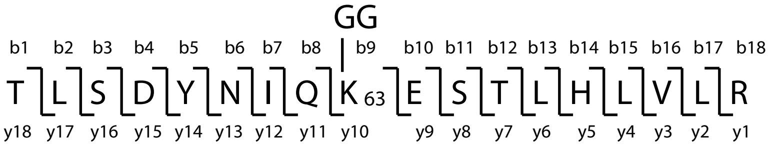

B

Precursor MS = 2244.1213 m/z

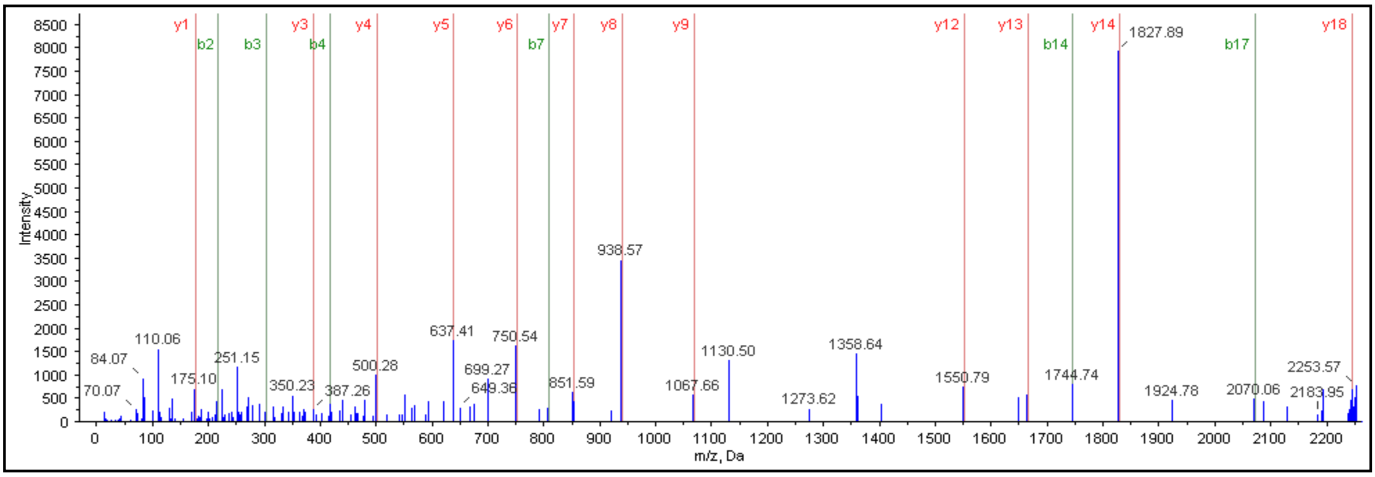

MS/MS (Sample #5, Parkin + Ubc13/Uev1, K63-linkage)

C

Precursor MS = 2244.1309 m/z

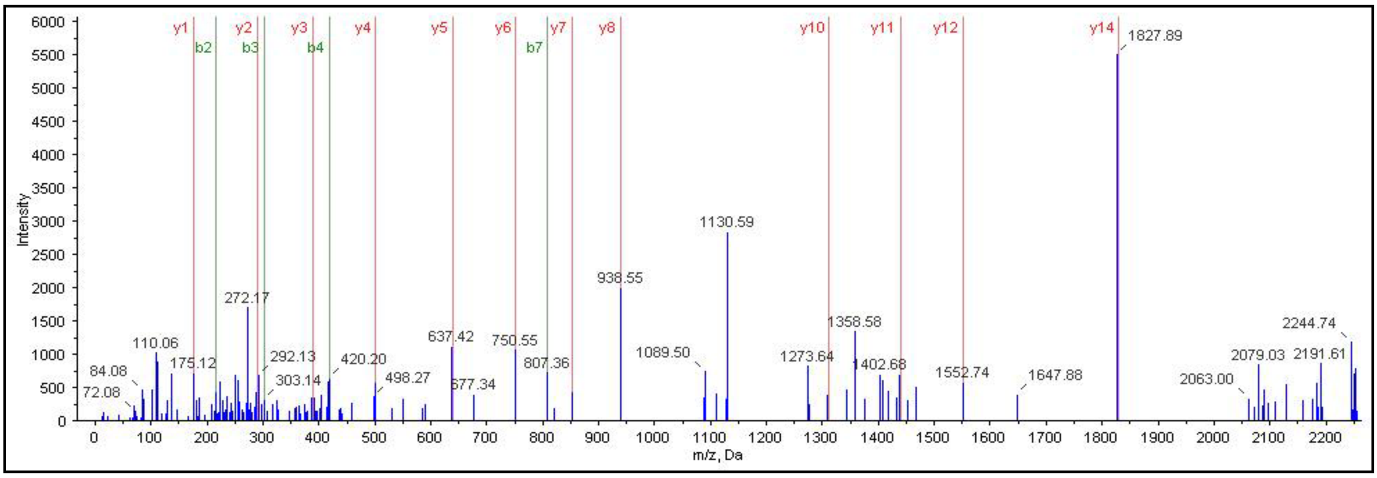

MS/MS (Sample #6, Parkin + Ubc13/Uev1, K63-linkage)

D

Precursor MS = 2243.8916 m/z

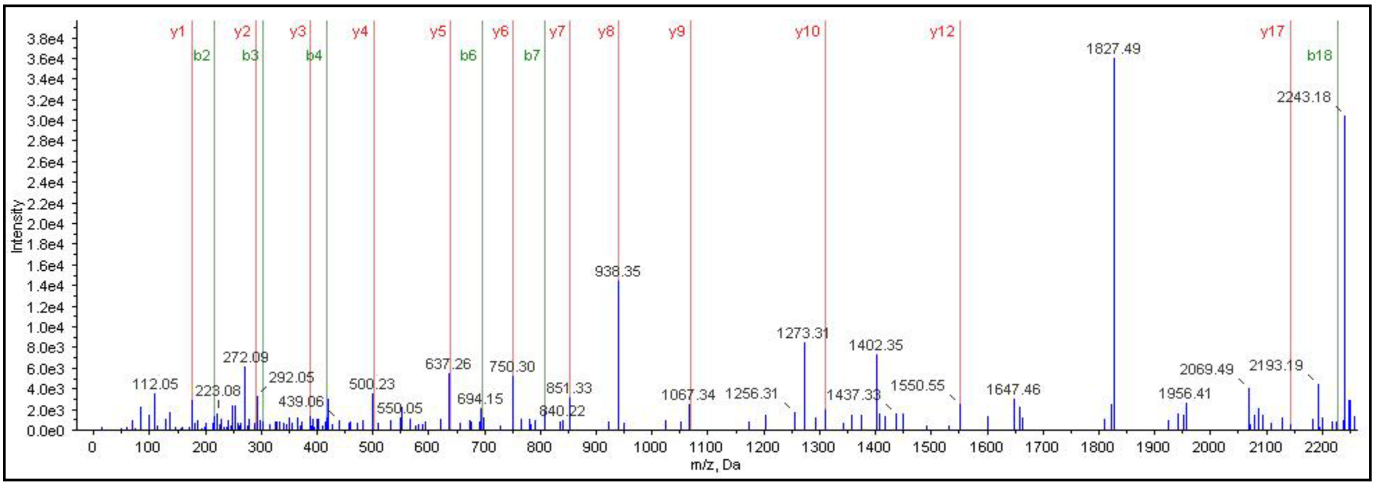

MS/MS (Sample #27, IBR-Ring2 + Ubc13/Uev1, K63-linkage)
